# Impact of progesterone on the immune system in women: a systematic literature review

**DOI:** 10.1007/s00404-023-06996-9

**Published:** 2023-03-18

**Authors:** Michelle Zwahlen, Petra Stute

**Affiliations:** Gynecologic Endocrinology and Reproductive Medicine, Department of Obstetrics and Gynecology, Inselspital Bern, University of Bern, Friedbuehlstrasse 19, 3010 Bern, Switzerland

**Keywords:** Immune system, Immune defence, Endogenous progesterone, Menstrual cycle

## Abstract

**Purpose:**

The immune system is influenced by many factors, including female sex hormones. The extent of this influence, however, is not completely understood so far. This systematic literature review aims at giving an overview of the existing concepts on how endogenous progesterone influences the female immune system along the menstrual cycle.

**Methods:**

The inclusion criteria were healthy female subjects in their reproductive age with a regular menstrual cycle. The exclusion criteria were exogenous progesterone, animal models, nonhealthy study populations and pregnancy. This led to 18 papers covered in this review. The search was performed using the databases EMBASE, Ovid MEDLINE and Epub, and the last search was conducted on September 18, 2020. Our findings were analyzed in four categories: cellular immune defense, humoral immune defense, objective and subjective clinical parameters.

**Results:**

We demonstrated that progesterone acts in an immunosuppressive way, favoring a Th-2-like cytokine profile. Further, we showed that progesterone inhibits mast cell degranulation and relaxes smooth muscle cells. Furthermore, we found supporting evidence for a so-called window of vulnerability after ovulation, where immune functions are lowered and mediated through progesterone.

**Conclusion:**

The clinical relevance of these findings is not completely understood yet. As the sample sizes of included studies were rather small and the content of them was broad, further investigations are needed to define to which extent the described changes actually clinically meaningful, whether they are capable of influencing the female health and how these findings can be used to increase well-being.

**Supplementary Information:**

The online version contains supplementary material available at 10.1007/s00404-023-06996-9.

## Introduction

During their reproductive lifespan, women experience monthly hormonal changes during the menstrual cycle. Ovarian estradiol (E2) production dominates the follicular phase, whereas progesterone (P) secretion peaks during the luteal phase [1, 2]. The hormonal fluctuations affect the whole female body, including the immune system. The immune system has to discriminate harmful from harmless. It is supposed to fight off infections, yet not to overreact and risk autoimmunity (reactions against self-antigens, e.g., against the body itself) [3]. Women are more prone to autoimmune diseases [4, 5] with the exact mechanisms not being fully elucidated. Furthermore, infectious diseases as revealed by the COVID-19 pandemic are still one of the world’s biggest health problems [6]. Thus the interaction between female sex hormones and the immune system is a relevant topic which has primarily been studied during pregnancy [7], but not during the menstrual cycle.

The aim of this systematic literature search was to investigate the impact of endogenous P (luteal phase of the menstrual cycle) on the female immune system, differentiating between the cellular and humoral immune system as well as between objective and subjective clinical signs and symptoms of the immune system.

## Materials and methods

To identify relevant literature, a systematic literature search was run following the PRISMA guidelines for systematic literature search. Search terms were sought out using the PICO method (population, intervention, comparison, outcome) and the search platform Ovid was scanned for according MeSH terms. The search was conducted on the platform Ovid. Search criteria (Appendix I Table 1) used were females, in the topics immunology and allergology, covering endogenous P or a broader term such as sex hormones or sex steroids, however, excluding E2 or testosterone, in a setting where its effect on the immune defense was described or where the terms immunosuppression or immunostimulation were used. A further criterium was an involvement of the menstrual cycle, excluding the topics delivery, pregnancy or birth. Synonyms for those terms were also included in the search. The only restriction was language wise, as only studies in English or German were taken into consideration. The search was conducted on July 27, September 10 and the last search was conducted on September 18, 2020. The databases used were Embase (1974–2020 September 18), Ovid MEDLINE(R) and Epub Ahead of Print, In-Process & Other Non-Indexed Citations, Daily and Versions(R) (1946 to September 18, 2020). Duplicates were removed by the search program and manually. The search led to 215 hits. For the detailed search strategy, see the Appendix II. The results were then imported to Mendeley as the reference program for this review.

Skimming through the titles one reviewer applied the criteria described above for eligibility. If unclear, the abstracts were scanned for relevance. Both reviewers then agreed on the selected papers, based on the content of their abstracts. Taken into consideration were all papers covering the link between endogenous P and the immune system or immunological factors, excluding studies on pregnancy, birth control or hormonal replacement therapy, as well as studies on animal models or on patients with a specific type of disease (i.e. nonhealthy subjects). The selection led to 20 hits of which 2 were secondarily excluded, as they were only available as a short abstract. The remaining 18 studies were then read, analyzed and used in this review.

The papers were scanned for information about the effect of P on humoral and cellular immunologic factors and objective as well as subjective differences in diseases and symptoms along the menstrual cycle. All findings were filled into a table, sectioned into the subtopics humoral changes, cellular changes, objective clinical changes and subjective clinical changes, as they were later used for the result chapter. The result chapter was then written using those notes.

## Results

### Overview of the studies’ characteristics

The literature search yielded 215 hits of which 20 publications fulfilled the inclusion criteria. As one article [8] was not available, and another one only had an abstract [9], the remaining 18 articles were included [2, 4, 5, 10–24] (Appendix I Table 2, Appendix III). All papers were published between 1997 [17] and 2016 [2]. Study designs comprised one PC-RCT [11], one head-to-head RCT [19], eight prospective cohort studies [2, 10, 12, 15–17, 24] [23], one cross-sectional cohort study [14], one retrospective cohort study [13], and one in vitro study [20]. In addition, five narrative reviews were identified [4, 5, 18, 21, 22]. Sample size ranged from 5 [17] to 61 [11] participants. Dropout rates were only reported by three studies [2, 10, 15] ranging from 14% [10] to 48% [15]. All 18 studies investigated the impact of endogenous P in premenopausal women. Subjects were generally healthy [2, 10–13, 15, 17, 23, 24], eumenorrhoeic [2, 10, 12–16, 19, 23, 24], non-smoking [12, 14, 15, 17, 19] and were not using hormonal contraception or other medication which might display an immunomodulatory effect [10, 12–17, 19, 23, 24]. Mean age was reported in ten articles [2, 10–14, 16, 19, 23, 24] and ranged from 20 [14] to 37 [16] years. Menstrual cycle phases were identified by different means, e.g. urinary LH surge [2, 10, 13, 19], or basal body temperature [19], respectively. Depending on the chosen endpoints, some trials used certain conditions, e.g., oral health [12, 15], HIV infection [16], sexual [10] or physical activity [14, 19, 23, 24], respectively. Six studies [10, 14, 16, 17, 19, 23] compared the target cohorts to other populations, e.g., males [17, 23], pregnant women [17], female athletes [19], HIV positive [16], amenorrhoeic [14], or sexually abstinent women [10], respectively. Five in 18 publications were interventional studies [11, 17, 19, 20, 23]. In detail, the only PC-RCT used a GnRH agonist as active comparator [11], two studies assessed the impact of physical exercise on immune factors [19, 23], one ex vivo in vitro study analyzed the impact of bacterial protein (lipopolysaccharide from salmonella minnesota) stimulation at different concentrations on subjects’ blood[17], and the only in vitro study used TD47 breast cancer cells treated with different substances (P, RU486, ZK98734, IL-1beta, TNFa) [20]. Duration of follow-up was generally short ranging from three hours [19] to three menstrual cycles [12, 24].

Many different endpoints were chosen apart from immune system markers, e.g. endocrine markers [4, 5, 10–15, 17–19, 21–23]. However, in this review, we will focus on immune system endpoints. The following laboratory endpoints were assessed: C-reactive protein (CRP [11, 13, 17]), glycodelin [13], secretory leucocyte protease inhibitor (SLPI [20]), elafin [20], lysozyme [23], lactoferrin [23], interleukins (IL-4 [10], IL-1beta [12, 15], IL-6 [17], IL-10 [16, 17]) and their receptors (IL-6 receptor [17]), other cytokines (TNF [12, 15, 17], IFN-gamma [10]), immunoglobulins (IgA [14, 23, 24]), white blood cell count (WBC [2, 11, 19]), T-cells [19] (regulatory [16], cytotoxic [16] or helper [16] T-cells), Th1/Th2 cytokine profile ratios [10], natural killer (NK) cells [19] and cell-mediated immunity (varizella zoster specific lymphocyte proliferative assay [16], natural cell-mediated cytotoxicity and lymphocyte proliferative response to mitogen [19]). Parameters were either measured in blood [2, 4, 11, 13–17, 19], saliva [10, 12, 14, 23, 24] (stimulated [14] or unstimulated [10, 12, 23, 24]), or gingival crevicular fluid [17, 20], respectively. Objective clinical endpoints of the oral immune system varied [12, 15], e.g., gingival bleeding index, modified gingival index and simplified oral health index or bleeding on probing, plaque index score and gingival index, respectively. Subjective clinical endpoints were assessed by questionnaires, diaries or logs for mood [24], menstrual cycle [19, 24], stressors or diseases [10, 14, 24], disgust sensitivity [2], depression [11], physical activity [24], sexual activity [10] or premenstrual (oral) symptoms [15]. Finally, of the narrative reviews included, three compared the impact of endogenous hormone exposure on the course of specific diseases, e.g., irritable bowel syndrome [22], allergies [5], or chronic non-infectious diseases [4].

### Impact of progesterone on the cellular immune system

The innate defence consists of cells with an unspecific and broad reaction toward foreign structures and molecules. Cells belonging to this group are macrophages, NK cells, dendritic cells and mast cells [3]. The adaptive immune defence consists of B-lymphocytes (B-cells) and T-lymphocytes (T-cells). Some B-cells, develop into plasma cells, responsible for the production of antibodies. T-cells differentiate into different subtypes, with specific tasks of their own. T-helper cells (CD4 + T-cells) for example, secrete cytokines, while T-killer cells (CD8 + T-cells) recognize and kill infected cells. As every individual B- or T-cell expresses a unique set of receptors, they act very selectively against pathogens, also explaining why the adaptive immune response takes more time to step into action than the innate response [3].

P exerts its effects mainly via membrane-bound and nuclear P receptors (PGR) [4, 18, 20, 22], and to a smaller extent also via glucocorticoid receptors (GR) [18]. PGR are expressed by various cell types and tissues including immune cells, e.g. mast cells [22], macrophages, dendritic cells, NK cells, CD4 + and CD8 + T-cells [4, 5, 21]. As PGR expression has also been found in thymus tissue, P is thought to have a direct impact on T-cell development and differentiation [18]. PGR regulation is complex. Two crucial mechanisms of PGR regulation are E2 inducing, and P downregulating PGR expression [18].

Our search identified 12 articles addressing the impact of P on the cellular immune system [2, 4, 5, 11, 16–23]. Apart from the five reviews [4, 5, 18, 21, 22], these were two RCT [11, 19], four prospective cohort studies [2, 16, 17, 23], and one in vitro study [20]. WBC count, a measure of all white blood cells, was shown to increase from follicular to luteal phase. During luteal phase, a correlation between serum P levels and WBC subtype count in blood was found. However, when comparing WBC changes along the timeline to P serum levels, no association was found [11]. In contrast, these results were not supported by another study, not finding any correlation between P and WBC at all [2].

Blood levels of IL-10 + T-helper cell (CD4 +) were shown to be higher during luteal than follicular phase. This effect was already noticeable in healthy subjects, but more pronounced in immunosuppressed (HIV-positive) subjects. In contrast, higher blood cytotoxic T-cell levels (CD8 Fox P3 +) during the luteal phase were only found in HIV-positive women [16]. This finding was supported by a significantly positive correlation between serum P levels and blood CD8 + T-cell counts in another study [19]. Accordingly, in the luteal phase, PGR alpha expression was upregulated in CD8 + T-cells, while PGR alpha expression in CD4 + T-cells did not change across the menstrual cycle [4]. Study results on blood CD4 + levels across the menstrual cycle are conflicting. The biggest and most recent study to date showed a decrease during luteal phase, while earlier studies did not observe changes in blood CD4 + levels across the menstrual cycle [4]. In respect to regulatory T-cells, scientific evidence supports a decrease during the luteal phase [4].

The total number of monocytes was shown to increase during the luteal phase in one source [4], while CD14 + and CD14 + /16 + cells (macrophages) blood levels did not change due to P serum levels in another paper [17].

Women generally present higher levels of mast cells in their tissues. P was shown to act as an inhibitor of mast cell degranulation [5, 22].

Studies on the impact of P serum levels on NK cell counts or on their activity were also conflicting, many of them not showing an effect [4, 19].

In summary, PGR are expressed by many tissues [4, 5, 21, 22], allowing P to influence cell numbers and/or activity. However, previous studies are inconsistent about the extent of this influence. The only consistent finding so far was an inhibition of mast cells by P [5, 22].

### Impact of progesterone on the humoral immune system

Antibodies and cytokines are part of the humoral immune system. Antibodies or immunoglobulins (Ig), produced by plasma cells, bind to foreign antigens, opsonizing (coating) them, or inducing immunologic reactions. Cytokines such as interleukins (IL), Interferons (IFN) and Tumor necrosis factors (TNF) can have inhibitory or inducing effects on immune cells. Those inducing immunologic effects similar to T-helper cells type 1 are called Th-1-like, while Th-2-like stands for T-helper cells type 2 like effects, i.e. those inhibiting immune responses [3, 10, 18]. Table 3 (Appendix I) shows a short overview of the molecules featured in this review.

Humoral immune factors were covered by 15 publications [2, 4, 10–15, 17–20, 22–24], including 2 RCT [11, 19], 7 prospective cohort studies [2, 10, 12, 15, 17, 23, 24], 1 cross-sectional study [14], 1 retrospective study [13], 1 in vitro study [20], and 3 reviews [4, 18, 22].

B-cell blood levels were stable across the menstrual cycle, making an effect of P on cell count rather unlikely. There was no consensus on a potential impact of P on the B-cell activity [4].

P was shown several times to induce a Th-2-like cytokine profile, suppressing the Th-1 like response [4, 18]. One study reported a rise in the ratio of Th-2-like cytokines to Th-1-like cytokines from follicular to luteal phase from 35 to 78%. Subjects with a Th-2-like cytokine ratio showed higher P-E ratios than those with a Th-1-like cytokine ratio [10].

Serum IL-1 was shown to decrease in the luteal phase, but E2 and P seemed to interact with each other and to have a biphasic effect on IL-1 serum levels. Low sex hormone serum levels increased IL-1 levels, while higher levels inhibited the secretion of IL-1 [4]. There were no consistent results for an impact of P on IL-1beta, a mediator for stress and inflammation, in blood [20, 22] and gingival crevicular fluid [12, 15], respectively. Similarly, results were inconsistent for P’s effect on serum IL-4 [4, 10] and IL-6 levels [4, 17]. IL-10 in healthy women was below the level of sensitivity throughout the menstrual cycle, thus an impact of P remains unclear [17].

Serum levels of TNFa, a proinflammatory cytokine [20], were shown to be either below the detection level across the menstrual cycle [17], or to increase during the luteal phase which might be a result of P serum level increase [4]. In gingival crevicular fluid, TNF alpha levels were either found to be stable across the menstrual cycle [15] or to be highest during the luteal phase but without showing a clear correlation with P serum levels [12].

IFN-γ showed a slightly negative correlation with serum P level, decreasing from ovulation to luteal phase. This effect was only noticed in sexually active women, but not in sexually abstinent women [10].

In vitro, P induced an increase in secretory leukocyte protease inhibitor (SLPI) mRNA and protein expression [20]. Both, the proof of the SLPI gene to have a progesterone response element (PRE) in its promotor region [20] and the observation that co-incubation with anti-progestogenic agents (e.g. RU486 and ZK98734) inhibited the described reactions, supported the initial hypothesis [20]. In contrast, elafin, also known to increase in the presence of proinflammatory cytokines, was not shown to have a PRE and its expression was not found to be altered by P making an impact of P unlikely [20].

Salivary soluble IgA (sIgA) levels were reported to be lower in amenorrhoeic compared to eumenorrhoeic women indicating that sex hormones may affect Ig production and secretion [14]. However, salivary sIgA levels were stable across the menstrual cycle [23, 24]. Thus, a clear association between P exposure and salivary sIgA has not been found yet [14].

Serum glycodelin levels, a placental protein produced by glandular epithelial cells of the endometrium during the luteal phase and early pregnancy, peaked about 1 week after ovulation and reached a nadir at about 4 days during the late follicular phase. There was a positive correlation between serum glycodelin levels 11–12 days after ovulation and the P serum levels 5–6 days after ovulation. At this point, the increase in serum glycodelin levels was the steepest, indicating that glycodelin-synthesis was taking place [13].

There were no menstrual cycle related changes in the acute phase protein CRP serum levels [11], and lysozyme or lactoferrin salivary levels[23], respectively.

In summary, Th-2 like cytokines increase with rising P serum levels [4, 18]. However, when assessing different types of cytokines, results varied largely between studies.

### Impact of progesterone on objective clinical parameters and scores

Overall, nine publications covered the impact of P on objective clinical parameters and scores [4, 5, 11, 12, 15, 16, 19, 21, 23]. Of those, two were RCT [11, 19], four prospective cohort studies [12, 15, 16, 23] and three narrative reviews [4, 5, 21].

P was found to have a relaxing impact on smooth muscle cells. Depending on the organ studied, this implies the following reactions: in blood vessels, dilatation lead to an increased blood flow and higher vascular permeability [12, 15], in the stomach, gastric emptying was slowed down [4], and within bronchioles, dilatation could increase airflow, thus reducing asthmatic symptoms, while a decrease in P levels could lead to acute exacerbation [4, 5]. P was also found to increase respiratory rates [4]. In the heart muscle, P seemed to shorten repolarization time, which was suggested to be protective for cardiac arrhythmia during the luteal phase [4].

Allergic diseases after puberty were found to be more common in females than in males. Evidence for a possible relationship between asthma or hay fever and irregular menses was shown. Atopic dermatitis was found to exacerbate around menstruation in some cases and so did asthma in the premenstrual phase, when P and E2 levels were dropping [4, 5].

Likewise, autoimmune diseases were reported to be more common in females. Some of them have been shown to worsen in the premenstrual phase, for example systemic lupus erythematosus and multiple sclerosis. In multiple sclerosis patients, P levels were within the normal range at all phases, but E2 levels were reduced, resulting in a high P–E ratio during the luteal phase, which correlated to bigger brain lesions [4]. In diabetic women, the impact of P on glucose tolerance is still unclear [4].

Dunbar et al. defined the term “window of vulnerability” [21]. It describes a time period of 7–10 days starting after ovulation, during which sex hormones are thought to suppress the innate cell-mediated immunity in a manner that make infections, such as HIV, more likely. It is hypothesized to be necessary for successful fertilization and implantation [21]. Similarly, Weinberg et al. found a linear decrease of cell-mediated immunity, lasting from late follicular to luteal phase [16]. Yet it remained unclear, whether this change could actually lead to a clinically relevant difference in disease susceptibility.

A few studies assessed the impact of sex hormones on inflammation in oral cavity [12, 15, 23]. In general, gingival crevicular fluid increases during inflammatory processes. In context of the menstrual cycle, gingival crevicular fluid secretion rate has been found to be higher when P serum levels are high [15].

Gingival bleeding on probing, another sign of inflammation, significantly increased from menstruation to P serum level peak during the luteal phase [15]. In contrast, both, the gingival bleeding index and the modified gingival bleeding index were positively correlated to ovulation, and there was a negative correlation between those two indexes and P salivary levels across the menstrual cycle [12].

In summary, P exerts a relaxing effect on smooth muscle cells, which implies an impact on the course of depending on disease type [12, 15]. Immunologic differences, were only recorded within the “windows of vulnerability”, when infection risk is higher, due to a decrease in cell-mediated immunity [16, 21].

### Impact of progesterone on subjective parameters

Overall, six studies addressed the impact of P on subjective parameters in study participants [2, 4, 14, 15, 22, 24].

In one study, premenstrual complaints were recorded in 44% of subjects; 22% had oral complaints, and 7% had aphthous lesions during menstruation, implying oral inflammation at this time of the menstrual cycle [15]. Similarly, in women with irritable bowel syndrome, increased pain and discomfort was reported during late luteal phase when P serum levels decline [22]. Women with rheumatoid arthritis suffered from increased morning stiffness and pain during menstruation and early follicular phase [4]. In an amenorrhoeic study cohort, more upper respiratory tract infections were reported compared to the eumenorrhoeic women [14]. However, feelings of disgust [2] and mood states did not alter across the menstrual cycle [24]. The same was true for training volume, type and intensity, according to training logs of enrolled subjects [24].

In summary, there was no clear association between subjective complaints and menstrual cycle phase although the premenstrual decrease of P serum levels was often associated with symptom worsening [15, 22].

## Discussion

The observation that P exerts an immunosuppressive effect is not new. However, previous studies focused on pregnancy. Thus, to our best knowledge, we are the first to address this subject in non-pregnant healthy women with regular menstrual cycle.

In our narrative review, we found that (1) P favors a Th-2-like cytokine profile, (2) P inhibits mast cell degranulation, a key mechanism of allergic reactions, (3) P increases vascular permeability via relaxation of smooth muscle cells, and (4) there is a “window of vulnerability” after ovulation, when immune functions are lowered, mediated through P.

The immunosuppressive effect is thought to be the result of a preferred release of Th-2-like cytokines [4, 10, 18]. Accordingly, an increase in the Th-2-like cytokine profile was found in the phases of higher P-E ratios, thus mainly during the luteal phase [10, 18]. This finding was supported by a reported IL-4 increase (one of the Th-2-like cytokines) proportional to P serum level increase during the luteal phase [4].

It would be expected that these effects are mirrored by the different Th-cell type counts as well. Unfortunately, most studies did not discriminate between type 1 and type 2 Th-cells, therefore, it is not possible to verify this thesis. Additionally, the findings were contradicting, which could be explained by the different types of Th-cells, summarized under one cell, going in opposite directions under changing P concentrations [4]. Only Weinberg et al. hinted at a similar effect of P on the Th-cells, as in their study the IL-10 + Th-cells (i.e. type 2 Th-cells) increased in the luteal phase, in accordance with the aforementioned results [16].

If we applied these findings to the current COVID-19 pandemic, it would imply that women were to be infected more often by SARS-CoV-2 than men, due to their inhibited immune response. In line with these expectations, infection rates have been shown to be higher in women aged 10–50 years (i.e. mostly in their fertile age) than in men of the same age. It, however, should be considered, that risks of exposure could also be a bias in this conclusion, as professions with a higher risk of infection, such as nurses, are often performed by females. Yet, as lethality of COVID-19 is likely caused by an exaggerated immune response, also known as the cytokine storm, the immunosuppressive effects of P could once again be part of the explanation, why mortality rates in women are lower than in men [25].

A further immunosuppressive effect is thought to be achieved by P decreasing PGR expressing mast cell degranulation potential [5, 22]. Inhibition of mast cell degranulation corresponds to an inhibition of local, or in more severe cases even systemic inflammation processes. This is important in allergic reactions, indicating a decrease in allergies after ovulation and an increase around menstruation. In the peri- and postmenopausal phase, where the female body must adapt to decreasing levels of female sex hormones, allergic diseases were shown to increase, supporting this thesis [5]. Yet, replacing sex hormones by, e.g. menopausal hormone therapy (MHT), has been shown to worsen allergic symptoms. This discrepancy may have various reasons, e.g. differences in endogenous and exogenously induced hormone levels and fluctuations, and types of E2 and progestogens used in MHT, respectively [5].

In our review, we further found a relaxing effect of P on smooth muscle cells, which can act out differently, depending on where the smooth muscle cells are located. In blood vessels it can lead to increased permeability and thus actually increase allergic reactions. In the lung, relaxing smooth muscle cells can be favorable for decreasing respiratory distress, mitigating asthma attacks [4]. However, different studies have shown a risk of asthma exacerbation during pregnancy, a time with even higher P serum levels [5]. In the digestive system, P can lead to fewer muscular contractions, resulting in constipation and delayed food passage [4, 22].

The term “window of vulnerability” was introduced to describe a time period, starting at ovulation and lasting for 7–10 days throughout the luteal phase, in which women are more prone to infection, due to a decreased cellular immune defense [21]. The physiological rational for this window is a support for successful fertilization and implantation of a semi-allogenic embryo. This process could otherwise be prohibited with a more active immune system, as the embryo could be recognized as foreign. Whether P is the key player of this effect, or whether E2 is involved as well or solely is not clear yet [21]. Nevertheless, this “window of vulnerability” falls together with the beforehand demonstrated increase in Th-2-like cytokines [4, 10, 18], known for being more immune tolerant, so an influence of P in this process seems likely.

Apart from these findings, results were contradictory in many cases. Furthermore, some topics had only been addressed by one researcher making comparisons impossible.

Accordingly, the clinical impact of the described effects of P on the immune system largely remain unknown. Large prospective studies monitoring P serum levels and recording infection rates would be helpful to learn more about the clinical relevance.

Our narrative review has some limitations. First, in contrast to research done in pregnancy, the number of studies investigating the impact of P on the immune system across the menstrual cycle was low. Secondly, study cohorts varied making it impossible to apply the results to all women. Also, study designs varied tremendously. For example, for menstrual cycle phase assessment, some authors used urinary LH tests [2, 10, 13, 19], basal body temperature assessment [19], or defined cycle phases based on hormone serum levels [15, 24]. Some studies used blood samples [2, 11, 13–17, 19], while others used saliva [10, 12, 14, 23, 24]or gingival crevicular fluids [12, 15], all three being completely different sources and having different objectives. While blood and serum assessments reflected the systemic immunologic state, saliva and gingival crevicular fluid represented only the local defense of the oral cavities. A solely local impact on the female reproductive tract was not covered by either of them. Endpoints also differed. As the “immune system” is a very broad and yet not fully understood target, future studies should focus on more specific, or even standardized endpoints. Finally, due to the interdependence of E2 and P, the discrimination between the unique effects of P and E2 on the immune system is almost impossible in an in vivo setting. Even when specifically focusing on the luteal phase of the menstrual cycle, when E2 serum levels are generally lower than P serum levels, their interaction cannot be neglected. Moreover, the P-E ratio was found to be more important than absolute values by some authors [4, 10]. Thus, the isolated effect of a sex steroid on immune cells does not always match the in vivo outcome due to their interaction. Eventually, this makes potential therapies of autoimmune diseases or prevention of infectious diseases not only very complex but also promising.

## Conclusion

The impact of P on the immune system is complex and still not thoroughly understood. We found that P does exert an effect on the immune system, which is in most cases immunosuppressive. The immunosuppressive actions are carried out through activation of Th-2-like cytokine pathways. More research is needed to assess the clinical relevance of these effects more thoroughly, to find potential implications in the treatment and prevention of diseases.

### Electronic supplementary material

Below is the link to the electronic supplementary material.

Supplementary file1 (DOCX 29 KB)

## Data Availability

As this is a systematic review there is no further data available.

## Appendix I: Tables

See Tables 1, 2, 3 here.

**Table 1:** Inclusion and exclusion criteria of the systematic literature search

Inclusion criteria	Exclusion criteria
Female human subjects	Animal models
Reproductive age	Exogenous progesterone
Endogenous progesterone	Pregnancy or HRT
Immune parameters	Disease-specific study cohorts
Measurements in blood and/or saliva	

**Table 2:** Brief overview of characteristics of included articles

	Review	Randomized controlled trial	Non-randomized controlled trial	Prospective cohort study	Cross-sectional study	Retrospective cohort study	In vitro study	Sample size (if stated)	Duration of follow-up in cycles	Dropout rate	Healthy	Eumenorrhoeic	Non-smokers	No medication	Age (if stated)	Blood samples	Gingival crevicular fluid	Saliva samples	Cellular endpoints	Humoral endpoints	Clinical endpoints	Subjective endpoints
Angstwurm et al.				X				5	2	–	X		X	X	22–34	X			X	X		
Weinberg et al.				X				20	1	–	X	X		X	18–45	X			X		X	
Baser et al.				X				27	1	48%	X	X	X	X	19–23	X	X			X	X	X
Shimizu et al.					X			8	–	–	X	X	X	X	Ø19	X		X		X		X
Bersinger et al.						X		36	1	–	X	X		X	20–32	X				X		
Khosravisamani et al.				X				27	3	–	X	X	X	X	Ø23		X	X		X	X	
Heede et al.		X						61	1	–	X				Ø24	X			X	X	X	
Lorenz et al.				X				30	1	14%	X	X			Ø23			X		X		
Burrows et al.				X				21	3	–	X	X		X	17–40			X		X		X
Gillum et al.			X					9	1	–	X	X			Ø22			X		X		
Żelaźniewicz et al.				X				30	1	40%	X	X			Ø28	X			X	X		X
Mulak and Taché	X							–	–	–	–	–	–	–	–	–	–	–	X			X
Dunbar et al.	X							–	–	–	–	–	–	–	–	–	–	–	X		X	
Jensen-Jarolim et al.	X							–	–	–	–	–	–	–	–	–	–	–	X		X	
Oertelt-Prigione	X							–	–	–	–	–	–	–	–	–	–	–	X	X	X	X
King et al.							X	–	–	–	–	–	–	–	–	–	–	–	X	X		
Bouillon et al.		X						14				X	X	X	Ø32	X			X	X		
Obendorf et al.	X							–	–	–	–	–	–	–	–	–	–	–	X	X		

**Table 3:** Overview of effects of featured humoral immune factors, inspired by the overview from Abbas et al. p. 303–306 [3]

Molecule	Secreted from	Function
IL-1	Macrophages, dendritic cells, endothelia	Attracts neutrophils and macrophages (chemotaxis), induces fever (via hypothalamus) [3]
IL-4	Th-2-lymphocytes, mast cells	Induces switch to IgE production in plasma cells and Th2-cell differentiation, alters macrophage activation [3]
IL-6	Macrophages, T-cells, endothelia	Induces production of acute phase proteins (liver) and proliferation of antibody-producing B-cells [3]
IL-10	Macrophages, T-cells, dendritic cells	Blocks cytokine production of macrophages and dendritic cells, inhibits co-stimulator-expression [3]
IFN-γ	NK cells, T-cells	Activates macrophages to digest phagocytosed pathogens, induces Th1-cell differentiation [3]
TNF-α	Macrophages, T-cells, mast cells	Exerts the same effect as IL-1 [3]
SLPI	Mucosal epithelia, leucocytes	Acts anti-microbial, inhibits proteases [20]
Elafin	Mucosal epithelia, leucocytes	Acts anti-microbial, inhibits neutrophil elastase and proteinase 3 [20]
Glycodelin (PP14)	Glandular epithelial cells	Acts immunosuppressive, not thoroughly understood yet [13]
C-reactive protein	Liver	Binds to pathogens (opsonization), thereby enables phagocytosis by macrophages [3]

*IFN* Interferon, *IgE* Immunoglobulin E, *IL* Interleukin, *SLPI* Secretory Leukocyte Peptidase Inhibitor *TNF* Tumor necrosis factor

## Appendix II: Search strategy

Database: Embase < 1974 to 2020 September 18 > , Ovid MEDLINE(R) and Epub Ahead of Print, In-Process and Other Non-Indexed Citations, Daily and Versions(R) < 1946 to September 18, 2020 > 

Search Strategy: 18.09.2020.

1. exp Women/(9309549)
2. (female or wom?n).ti,ab. (4270886)
3. 1 or 2 (11362839)
4. exp “Allergy and Immunology”/(367875)
5. (((impact or influence or effect or defen?e) adj3 immun*) or immunosuppress* or immunostimulat*).ti,ab. (477429)
6. 4 or 5 (829499)
7. exp Progesterone/(158875)
8. (((progesteron* or “sex hormone*” or “sex steroid*” or “steroid hormone*”) adj3 endogenous) or exogenous).ti,ab. (323316)
9. (Progesteron* not MPA not medroxyprogesterone not “medroxyprogesterone acetat*” not estrogen not estradiol not oestrogen not testosteron*).ti,ab. (76191)
10. 7 or 8 or 9 (501752)
11. exp Menstrual Cycle/(143107)
12. ((menstrua* or “ovarian cycle*” or ‘endometrial cycle*’) not delivery not pregnancy not birth).ti,ab. (88552)
13. 11 or 12 (187010)
14. 3 and 6 and 10 and 13 (230)
15. remove duplicates from 14 (220)
16. limit 15 to (english or german) (215)

## Appendix III: Supplementary file

Supplementary file 1: Overview of included articles.
