# Supplementary material for: Impact of progesterone on the immune system in women: a systematic literature review
Source: Arch Gynecol Obstet. 2023 Mar 18;309(1):37–46. doi: 10.1007/s00404-023-06996-9 (PMC10024519; doi:10.1007/s00404-023-06996-9)
Supplement: Supplementary file 1 — Supplementary file1 (DOCX 29 KB) [file 404_2023_6996_MOESM1_ESM.docx]

| Author, Journal & Year | study design | study cohort (n=...) | study cohort characteristics (sex, age, etc.) | intervention | duration of follow-up | primary endpoint | tool to assess primary endpoint | secondary endpoints | tools to assess secondary endpoints | relative risk unadjusted | relative risk adjusted |
| --- | --- | --- | --- | --- | --- | --- | --- | --- | --- | --- | --- |
| M. W. A. Angstwurm, R. Gärtner, H. W. Löms Ziegler-Heitbrock, Cyclic Plasma IL-6 Levels during normal menstrual cycle, 1997 | prospective cohort study | n= 19 (5 with normal cycles, 5 pregnant, 9 men) | healthy (according to History, physical examination and laboratory), non-smokers, no allergies, regular lifestyle, no medication. Women aged 22-34. (Control group pregnant women in weeks 19-32; men were 24-35years old) | in vitro: stimulation of peripheral whole blood with LPS from Salmonella minnesota | at least two cycles per participant | cycle-changes in IL-6, IL-6 receptor and TNF plasma levels correlating with sex hormone levels | blood and plasma at days 3-5, 10, 14, 21 and 28 (in pregnant women and men at least two samples with interval of 1-2 weeks). TNF, IL-6 and IL-10 measured with ELISA | changes in those cytokines after ex vivo stimulation with LPS | whole heparinized blood, incubated 4h, stimulated with different concentrations of LPS (1,10 and 1000ng/ml), used as whole blood culture system and analysed along a cycle | n.a. | n.a. |
| A. Weinberg, L. Enomoto, R. Marcus, J. Canniff, Effect of menstrual cycle variation in female sex hormones on cellular immunity and regulation, 2010 | prospective cohort study | n = 40 (20 healthy and 20 HIV infected women) | aged 18-45 (mean age 37), healthy women: regular menses, previous Varizella Zoster Virus infection in history and anti-VZV-antibodies. No pregnancy, no hormonal contraceptives, no immunosuppressive therapy or other disease. HIV infected: CD4+ counts over 300 cells/Mikroliter or >20% CD4+ cells and a plasma HIV RNA <10'000 copies/mL | none | one cycle | variation in T-cell plasma concentration | blood sampling on days 1-4, 10-14 and 20-24 (i.e. early and late follicular and luteal phase) for oestradiol, progesterone and Treg cell titers. Treg cell titers measured using peripheral blood mononuclear cells: CD4+ and CD8+ T-cells (isolated from blood), stained according to cell-markers and then counted. | differences in cell mediated immunity | Varizella zoster virus-cell mediated immunity measured with the lymphocyte proliferation assay. Peripheral blood mononuclear cells were obtained from blood samples (via centrifugation), treated with VZV-infected cell lysate and then Thymidine incorporation was measured | n.a. | n.a. |
| U. Baser et al, Gingival Inflammation and Interleukin 1-beta and Tumor Necrosis Factor-Alpha Levels in Gingival Crevicular Fluid during the Menstrual Cycle, 2009 | prospective cohort study | n = 27 | female white dental students, aged 19-23years, regular menses, no smoking, no pregnancy, no oral contraceptive pills, no fertility treatment, no gingival recession, signs of peridontitis, fixed or removable prosthesis or irregular alignement of teeth in the oral examination, healthy, no history of medication influencing the periodontal tissues and no antibiotics in prior 6 months | none | one cycle | correlating differences in GCF, IL-1β, TNF-α in GCF and blood sex hormone levels | blood and GCF sampling at menstruation (defined as day with lowest sex hormone levels), ovulation (defined as oestrogen peak) and progesterone secretion day (defined as progesterone peak). Collection of GCF with a filter paper strip inserted until resistance felt, left for 30seconds. Discharged, if contaminated with blood. Analysis with ELISA method. Measured were total IL-1beta levels and concentration in GCF. | clinical and subjective differences in oral health along cycle | subjective and clinical evaluation at menstruation (defined as day with lowest sex hormone levels), ovulation (defined as oestrogen peak) and progesterone secretion day (defined as progesterone peak). Patients asked about oral (edema, discomfort, ichtiness of gingiva, aphthous lesions) or systemic complaints. Intraoral examination: bleeding on probing (percentage of sites bleeding within 30 sec), plaque index score and gingival index | n.a. | n.a. |
| K. Shimizu et al., Mucosal immune function comparison between amenorrheic and eumenorrheic distance runners, 2012 | crosssectional study | n= 21 (amenorrheic = 13; eumenorrheic = 8) | All: japanese female collegiate endurance runners, no drugs affecting the immune system taken, no tobacco. eumenorrheic: mean age 19,9+/-0,8 years, 10-14 cycles per year (in prior year) / amenorrheic: mean age 20+/- 0,3 years, fewer than 4 cycles in prior year | none | no follow up, only a sampling once | differences in secretory IgA, saliva secretion correlating to sex hormone changes | saliva and blood samples collected in morning. Saliva: collected in morning, no breakfast, mouth rinsed, then rested, then stimulated saliva secretion by chewing a cotton ball for 2min. Saliva was then centrifuged and frozen until analysis of salivary IgA amount, using ELISA method. Calculation of IgA secretion rate. Blood samples were collected afterwards. There serum sex hormone levels were measured using radioimmunoassay. | upper respiratory tract infection symptoms | patients reported symptoms by filling out a questionnaire. Subjects were asked about URTI symptoms such as sore throat, headache, fatigue, emesis, cough, runny nose or fever during the last month. | n.a. | n.a. |
| N. Bersinger et al., Serum glycodelin pattern during the menstrual cycle in healthy young women, 2009 | retrospective study | n= 36 | healthy women aged 20-32 (mean 25,5+/-5,5years), regular menstrual cycles (25-31days), BMI in normal range (mean: 21,9+/-3,1kg/m^2), no oral contraceptives or other medications in prior 3 months, biphasic pattern of basal body temerpature, normal Gynecological ultrasound, papanicolaou cervical smear and pre treatment hormonal values (FSH, LH, Prolactin, E2, free testosterone and dehydro-epiandrosterone sulfate) | none | one cycle | serum glycodelin level in luteal phase and sex hormone levels | blood samples in morning, every second day from menstruation for 8 days, then every day until 4 days after ovulation (controlled with a urinary LH-detection test), then again every second day until the next menstruation onset (mean 16 samples per patient). Blood was allowed to clot, centrifuged and then stored in frozen fashion. Glycodelin was analysed with a microplate enzyme immunoassay. | differences between follicular phase serum glycodelin in study subjects and patients with stimulated cycles due to IVF | blood sampling daily or every second day for a cycle an analysis as described in tool to assess primary endpoint, but compared to values of IVF recipients (n =51) | n.a. | n.a. |
| Khosravisamani M, Maliji G, Seyfi S, Azadmehr A, Abd Nikfarjam B, Madadi S, Jafari S. Effect of the menstrual cycle on inflammatory cytokines in the periodontium, 2014 | prospective cohort study | n =27 | females, mean age 23,72, duration of the cycle 28+/-3d and duration of the menstruation 5-7d, no use of contraception or other drugs affecting sex hormones, no antibiotics or NSAIDs 3 months before trial start, not pregnant/breastfeeding, no history of systemic diseases, peridontitis, gingivitis, canker sores, inflammatory mouth lesions, severe dental crowding. no alcohol or tobacco. no scaling, root planing or periodontal tissue surgery in last 6 months, no dental prostheses or orthodontic applicances. | none | three menstural cycles | changes in IL-1β, TNF-α in GCF along cycle | Unstimulated saliva samples collected per spitting method and GCF, centrifuged, frozen, and analysed for Progesterone and estrogen, only when all samples had been collected to determine the stages of the cycle. sampling took place at menstruation (days 1-2), ovulation (days 12-14) and premenstruation (days 22-24). GCF samples collected in morning from mesiobuccal gingival sulcus of the first and second maxillary molars, with paper points (the creviculars were airdried before). Paper points were collecting for 30 seconds. If contaminated with saliva or blood, they were not used. Cytokines were analysed with ELISA | clinical changes in oral health | gingival bleeding index (GBI), modified gingival index (MGI) and simplified oral index (recorded at same timepoints) | n.a. | n.a. |
| M. Heede et al, Sex hormone fluctuation triggers subclinical depressive symptoms and affects immune cell numbers in peripheral blood in healthy women, 2013 | double blinded RCT | n = 61 | healthy women, mean age 24,3+/-5 years | GnRHa (Gonadotropin releasing-hormone Agonist) or placebo | one cycle | correlation between sex hormone manipulation with GnRHa and WBC | blood drawn at baseline (cycle day 6,6+/-2,1) and follow up (16,2+/-2,6 days after intervention). In 40 participants two more samples were collected on the intervention day (cycle day 22,7+/-2,7) and 3-5 days after expected stimulatoin phase of GnRHa. Blood was analysed for CRP, white blood count (and differentiated between lymphocytes, monocytes and neutrophils) as well as sex-hormone analyses | differences in subclinical depressive symptoms | subclinical depressive symptoms were analysed with a Hamilton 17-item scale. | n.a. | n.a. |
| Tierney K. Lorenz et al. Sexual activity modulates shifts in Th1/Th2 cytokine profile across the menstrual cycle: an observational study, 2015 | prospective cohort study | n = 30 (sexually abstinent = 16, sexually active = 14 ) | both groups healthy premenopausal women, mean age 23,44 years, regular menstrual cycle of 26-34 days, no hormonal or immunoactive medications or other medications except for vitamin or herbal suppelements used occasionally (maximum once a week) or over the counter antihistamines or analgesics (those were allowed). no pregnancy or lactation in last 12 months and no medical condition that could affect immune response. Sexually abstinent: no partnered genital sexual activity in last 4 months. Sexually active: penile-vaginal intercourse at least once a week and with only one partner | none | one cycle | difference in changes in TH1/Th2 cytokine profile between sexually active and abstinent women | saliva samples at four time points (menstrual, follicular, ovulatory and luteal phase) analysed with ELISA for Estrogen, Progesterone, IFN-gamma and IL-4. Timepoint one: withing 2 days of menstrual bleeding onset. Two: 7-10 days after menstruation onset. Three: within 48h of ovulation, which was calculated and tested with LH urine test stripes and four: 7-10days after ovulation. 1 and 4 were made at laboratory 2 and 3 at home. Unstimulated saliva samples through passive drool. | association between P-E ratio and cytokine ratio | statistical analysis of salivary results | n.a. | n.a. |
| M. Burrows, S. Bird and N. Bishop, The menstrual cycle and ist effect on the immune status of female endurance runners, 2002 | prospective cohort study | n = 20 | highly trained, female endurance runners, eumenorrheic, aged 17-40years (mean 27+/-10), currently competing at distances from 1,5-42km, no oral contraceptive users. No illnesses or drug use in previous 6 weeks or reproductive disorders. Average menstrual cycle length of 29+/-7 days. Average luteal phase lengt 13+/-2 days. | none | 3 cycles | levels of salivary P, salivary IgA concentation, IgA secretion rate and saliva secretion rate at different time points | Unstimulated Saliva collection daily (with cotton swabs sublingual) for three months (first two only for Progesterone, to identify luteal phase, third month progesterone and IgA); samples were analyzed using ELISA. IgA analysis was used to calculate saliva flow rate, IgA secretion rate and IgA concentration | none | n.a. | n.a. | n.a. |
| T. Gillum et al, The effects of exercise, sex and menstrual phase on salivary antimicrobial proteins, 2014 | prospective cohort study | n = 18 (9 women and 9 men) | men and women: no cardiovascular, pulmonary or metabolic diseases, no illnesses in last 3 months, recreationally active. Females: eumenorrheic with cycles of 28-32 days, no hormonal contraceptives in last 6 months, mean age 22,3+/-2,4years (vs. men 21,1 +/-1,1 years) | 45min treadmill running at 75% VO2 peak | one cycle (two trials per person) | impact of exercise on salivary immune factors | Unstimulated saliva. At baseline VO2 peak assessed individually. Aimed at calculating 75% VO2 peak (expressed per ml of fat free body mass for comparability). treadmill running for 45 min (heart rate checked every 5 min). unstimulated passive drool saliva samples before exercise, right after the 45min exercise and 1h post-exercise. 2 trials/participant. For females one in follicular, on in luteal phase. Men were matched to the women and had approx. the same time apart two measures. Saliva: ELISA | none | n.a. | n.a. | n.a. |
| A. Żelaźniewicz et al., The progesterone level, leukocyte count and disgust sensitivity across the menstrual cycle, 2016 | prospective cohort study | n = 30 | healthy women in reproductive age (mean age 28,9years), detectable ovulation (positive LH test), no reported infection and normal values of Progesterone | none | one cycle | variation in disgust sensitivity along menstrual cycle correlating with P levels | Blood extraction (P and WBC) and Disgust sensitivity with two questionnaires: 1) Disgust-Scale revised (core disgust, animal reminder and contamination disgust) and 2) two parts of the three-domain disgust scale (pathogen disgust and moral disgust). Two measures: one in menstruation (day 2-4 within start of bleeding), one in mid-lutal phase (6-8 days after ovulation, determined with a LH urinary test. P: ELISA | WBC changes along menstrual cycle | blood extractions were analysed for WBC using a hematology analyzer with impedance variation | n.a. | n.a. |
| A. Mulak and Y. Taché, Sex difference in irritable bowel syndrome: do gonadal hormones play a role?, 2010 | review | - | - | - | - | correlation between sex hormones and IBS manifestation | tools were not reported | none | n.a. | n.a. | n.a. |
| B. Dunbar, M. Patel et al. Endocrine control of mucosal immunity in the female reproductive tract: Impact of environmental disruptors, 2012 | review | - | - | - | - | infections in female reproductive tract | tools were not reported | none | n.a. | n.a. | n.a. |
| E. Jensen-Jarolim, E. Untersmayr, Gender-medicine aspects in allergology, 2008 | review | - | - | - | - | susceptibility to allergic diseases in relation to sex hormone changes | tools were not reported | hypersensitivity towards own sex hormones | not stated | n.a. | n.a. |
| S. Oertelt-Prigione, Immunology and the menstrual cycle, 2011 | review | - | - | - | - | changes in immunologic mediators correlating to sex hormone levels | tools were not reported | clinical changes in chronic diseases along the menstrual cycle | not stated | n.a. | n.a. |

Supplementary file 1: Overview of included articles.

Abbreviations

BMI Body mass index

CD4+ cells/Th-cell T-helper cells

CD8+cells Cytotoxic T-cells

CRP C-reactive protein

E2 Estradiol

ELISA Enzyme-linked immunoassay

FSH Follicle stimulating hormone

GBI Gingival bleeding index

GCF Gingival crevicular fluid

GnRHa Gonadotropin releasing Hormone Agonist

HIV Human immunodeficiency virus

IBS Irritable bowel syndrome

IgA Immunoglobulin A

IL Interleukin

IVF In vitro fertilization

LH Luteinizing hormone

LPS Lipopolysaccharids

MGI Modified gingival index

n.a. Not applying

NSAIDs Non-steroidal anti-inflammatory drugs

P Progesterone

P-E ratio Progesterone/Estrogen ratio

RCT Randomized controlled trial

TNF Tumor necrosis factor

Treg cells Regulatory T cells

URTI Upper respiratory tract infection

VO2 max Maximal oxygen uptake

VZV Varizella zoster virus

WBC White blood cells
